# Hyperleptinemia positively associated with central arterial stiffness in hemodialysis patients

**DOI:** 10.1371/journal.pone.0190694

**Published:** 2018-01-05

**Authors:** Chiu-Huang Kuo, Yu-Li Lin, Chung-Jen Lee, Chih-Hsien Wang, Yu-Hsien Lai, Hung-Hsiang Liou, Bang-Gee Hsu

**Affiliations:** 1 Division of Nephrology, Buddhist Tzu Chi General Hospital, Hualien, Taiwan; 2 Department of Nursing, Tzu Chi University of Science and Technology, Hualien, Taiwan; 3 School of Medicine, Tzu Chi University, Hualien, Taiwan; 4 Division of Nephrology, Department of Medicine, Hsin-Jen Hospital, New Taipei City, Taiwan; Hospital Universitario de la Princesa, SPAIN

## Abstract

**Objective:**

Leptin plays a role in stimulating vascular inflammation, vascular smooth muscle hypertrophy, and augmenting blood pressure, which contributes to the pathogenesis of atherosclerosis and leads to arterial stiffness. This vascular damage, measured by carotid-femoral pulse wave velocity (cfPWV), is recognized as an independent predictor of cardiovascular mortality in hemodialysis (HD) patients. The aim of this study was to evaluate the relationship between serum leptin and arterial stiffness in HD patients.

**Patients and methods:**

In 112 of the 126 HD patients were eligible and their biochemical data were collected for analysis. Serum leptin level was measured using a commercial enzyme-linked immunosorbent assay kit. Carotid-femoral pulse wave velocity was measured by a validated tonometry system (SphygmoCor). Those have cfPWV values above 10 m/s are defined as the high arterial stiffness group.

**Results:**

Among the participants, thirty-eight of them who were in the high arterial stiffness group, had a higher prevalence of diabetes mellitus (p = 0.002), age (p = 0.029), body mass index (BMI, p = 0.018), body fat mass (p = 0.001), hemoglobin (p = 0.040), and serum leptin levels (P<0.001). Multivariable logistic regression analysis showed that leptin (odds ratio [OR] 1.09; 95% confidence interval [CI] 1.04–1.14; p <0.001), diabetes (OR 7.17; CI 1.39–37.00; p = 0.019), body fat mass (OR 1.16; CI 1.02–1.33; p = 0.027); and hemoglobin (OR 2.11; CI 1.15–3.87; p = 0.015) were independently associated with arterial stiffness in HD patients.

**Conclusion:**

In our study, hyperleptinemia was positively correlated to the high cfPWV and thus was related to high arterial stiffness in HD patients.

## Introduction

Advanced chronic kidney disease provides several mechanisms responsible for the exacerbation of cardiovascular disease, which includes the activation of the renin-angiotensin system, oxidative stress, elevated asymmetric dimethylarginine (ADMA), inflammation, dyslipidemia and vascular calcification. Vascular calcification, caused by increased calcium and phosphate loading, can lead to arterial stiffness. Carotid and aortic stiffness independently predict death in adult patients with end-stage renal disease (ESRD) [1–2]. In fact, this accelerated cardiovascular disease accounted for more than one -half of death in these patients. Therefore, it is important to explore this central arterial stiffness in ESRD patients. Carotid-femoral pulse wave velocity (cfPWV) is applied as one of the measurements for arterial stiffness and higher pulse wave velocity value predicts poor cardiovascular outcomes [3–4].

Leptin, one of the adipokines, is known to regulate various physiological processes including neuroendocrine functions, appetite, energy expenditure, glucose homeostasis, and insulin sensitivity. Recently, by increasing sympathetic tone and thus elevating blood pressure, leptin was found to be related to atherosclerosis in patients with cardiovascular disease [5]. Furthermore, through the inhibition of endothelial nitric oxide synthase, leptin was also related to coronary artery calcification in non-diabetic individuals [6]. However, this correlation between serum leptin level and arterial stiffness was uncertain in ESRD patients.

Therefore, this study is aimed to assess the association between the serum leptin level and arterial stiffness in hemodialysis (HD) patients.

## Materials and methods

### Patients

Patients older than 20 years of age, who underwent three HD sessions each week for at least six months at a single medical center in Hualien, Taiwan, were recruited between March and July in 2015. The study was conducted in accordance with the Declaration of Helsinki and was approved by the Protection of Human Subjects Institutional Review Board of Tzu-Chi University and Hospital. The approval number was IRB103-136-B. Informed written consent was obtained from all patients prior to their enrollment in this study. The estimating sample size should be more than 108 to meet the power of 90%. Participants suffered from acute infection, acute myocardial infarction, or pulmonary edema at the time of blood sampling were excluded.

### Anthropometric analysis

Before dialysis session, bioimpedance measurement of fat mass was performed at the bedside according to the standard tetrapolar whole body (hand-foot) technique, using a single-frequency (50-kHz) analyzer (Biodynamic-450, Biodynamics Corporation, Seattle, USA). Body height was measured to the nearest half centimeter. Waist circumference was measured at the shortest point below the lower rib margin and the iliac crest to the nearest half centimeter. The post-HD body weight was recorded by half-kilogram with the patient in light clothing and without shoes after HD. The weight (kilograms) divided by height squared (meters) yielded body mass index (BMI). All the measurements were carried out by the same operator. Percentage of fat mass was analyzed by specific formula supplied by the manufacturer [7–10].

### Biochemical measurement

Approximately 0.5 mL of blood samples were drawn for hemoglobin and white blood cells counts (Sysmex K-1000, Sysmex American, Mundelein, IL, USA) and the rest of 4.5 mL were immediately centrifuged at 3000 × g for 10 min for biochemical analyses. Within one-hour of collection, serum samples were stored at 4°C. Blood urea nitrogen, serum creatinine, glucose, total cholesterol, triglyceride, iron, total iron-binding capacity, ferritin, total calcium, and phosphorus were measured by using an autoanalyzer (COBAS Integra 800, Roche Diagnostics, Basel, Switzerland); serum leptin concentration by a commercially available enzyme immunoassay (EIA; SPI-BIO, Montigny le Bretonneux, France) [10]. Serum intact parathyroid hormone levels (iPTH) were tested using an enzyme-linked immunosorbent assay (ELISA; Diagnostic Systems Laboratories, Webster, Texas, USA) [9–10].

### Carotid-femoral pulse wave velocity (cfPWV) measurement

As previously described, after 10 minutes rest in a quiet, temperature-controlled room, the participant was under a supine position during the measurement [7–10]. We applied a pressure tonometer (SphygmoCor system, AtCor Medical, Australia) to measure cfPWV between carotid and femoral sites and to record the pressure pulse waveform in the underlying artery transcutaneously. The integral software taped each set of the pulse wave and electrocardiography (ECG) data and calculated the mean time differences between the R-wave. Records were documented simultaneously with an ECG signal, which provided an R-timing reference. This measurement took ten consecutive cardiac cycles in average. The cfPWV was calculated by distance and the mean time difference between the two recorded arterial sites. Quality indices, included in the software, were constructed to ensure the uniformity of data. In this study, patient whose cfPWV values above 10 m/s is defined as high arterial stiffness group, according to the European Society of Hypertension and the European Society of Cardiology (ESH-ESC) 2013 Guidelines [11].

### Statistical analysis

Data were tested for normal distribution using the Kolmogorov-Smirnov test. Data were expressed as mean ± standard deviation (SD) for normally distributed data and data were expressed as median and interquartile ranges for non-normal distributed data. Comparisons between patients were performed using the Student’s independent t-test (two-tailed) for normally distributed data, or the Mann-Whitney U test for parameters that presented a non-normal distribution. The glucose, ferritin, triglyceride, iPTH, leptin and HD duration datasets showed skewed non-normal distributions and therefore these were recalculated by transformation to the logarithm to the base 10; after this transformation the log-glucose, log-ferritin, log-triglyceride, log-iPTH, log-leptin, and log-HD duration then became normally distribution. Data expressed as the number of patients was analyzed by the χ2 test. Clinical variables that correlated with log-leptin levels in HD patients were evaluated by univariable linear regression analysis. Variables that were significantly associated with arterial stiffness in HD patients were tested for independence by multivariate logistic regression analysis. All statistical analyses employed SPSS for Windows (version 19.0; SPSS Inc., Chicago, IL, USA). A p-value < 0.05 was considered statistically significant.

## Results

**Fig 1:**
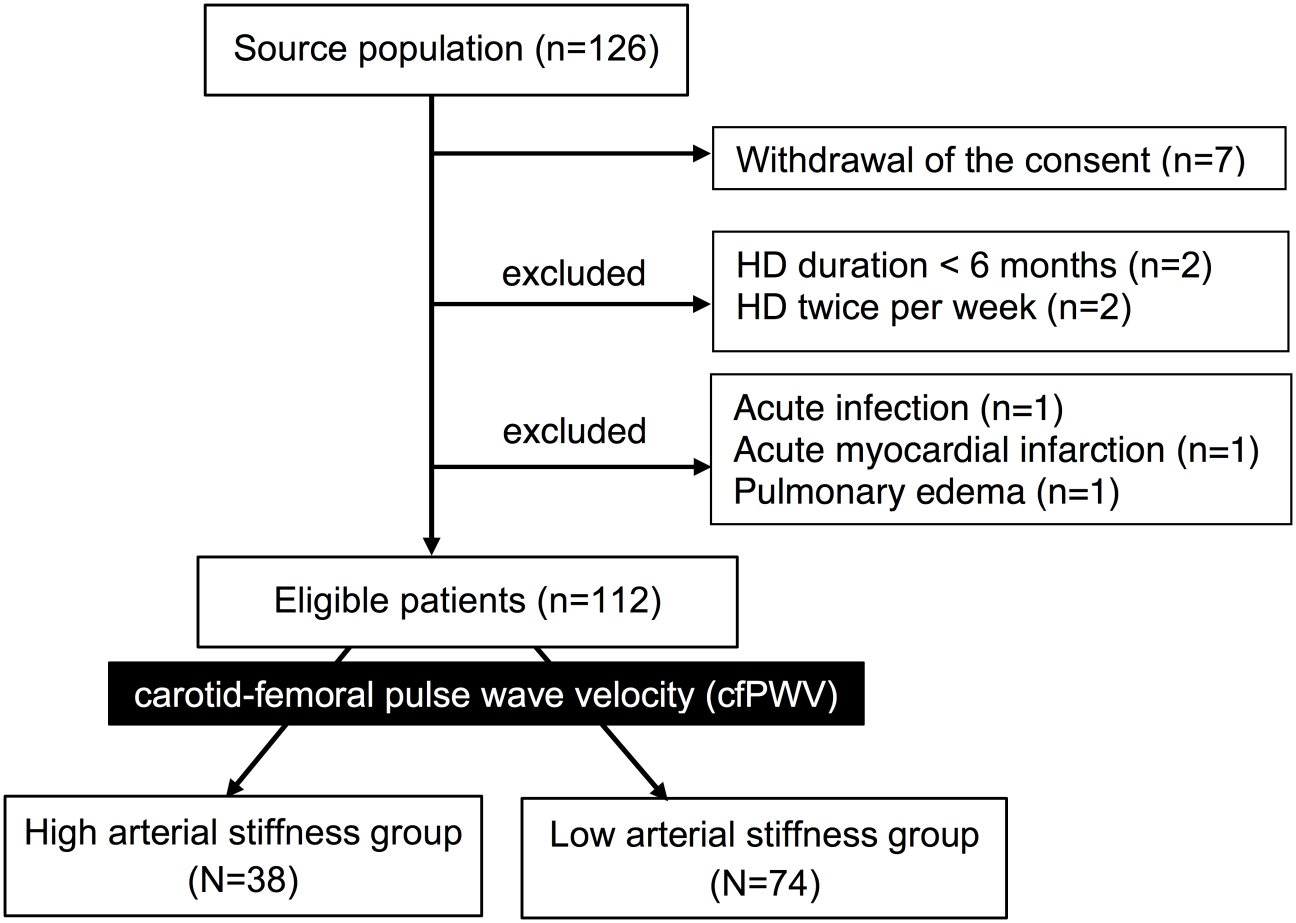
Study design.

A total of 126 HD patients were enrolled, and 14 of them were excluded on account of withdrawing consents (N = 7), HD duration < 6 months (n = 2), HD twice per week (n = 2), acute infection (n = 1), acute myocardial infarction (n = 1), and pulmonary edema (n = 1). In 112 HD patients, 57 males and 55 females, were eligible for this study (Fig 1). Their mean age was 62.54 ± 13.52 years, 45 patients (40.2%) had diabetes and 58 patients (51.8%) had hypertension. The median HD duration was 4.63 (1,80–10.32) years. Clinical and laboratory characteristics of the HD patients are shown in Table 1. Thirty-eight patients (33.9%) were defined as high arterial stiffness, while 74 patients (66.1%) as low arterial stiffness. Compared to the low arterial stiffness group, the high arterial stiffness group had older age, higher post dialysis body weight, BMI, waist circumference, and body fat mass. Laboratory data included hemoglobin, triglyceride, and serum leptin levels were also significantly higher in the high arterial stiffness group. The calcium, phosphate, and intact PTH levels had no significant difference between two groups.

**Table 1:** Clinical variables of the 112 hemodialysis patients with high or low arterial stiffness.

Characteristics	All Patients (n = 112)	Low Arterial Stiffness Group (n = 74)	High Arterial Stiffness Group (n = 38)	P
Age (years)	62.54 ± 13.52	60.54 ± 13.48	66.42 ± 12.92	0.029*
HD duration (years)	4.63 (1.80–10.32)	4.74 (1.79–11.21)	4.55 (1.86–6.92)	0.325
Post-HD body weight (Kg)	61.91 ± 14.90	59.80 ± 15.28	66.02 ± 13.39	0.036*
Waist circumference (cm)	91.35 ± 13.06	88.77 ± 12.88	96.37 ± 12.05	0.003*
Body mass index (Kg/m^2^)	24.10 ± 4.96	23.31 ± 5.02	25.64 ± 4.52	0.018*
Body fat mass (%)	27.73 ± 6.51	25.79 ± 6.55	31.49 ± 4.54	0.001*
Carotid-femoral PWV (m/s)	9.49 ± 3.42	7.52 ± 1.58	13.32 ± 2.71	<0.001*
Aortic SBP (mmHg)	131.47 ± 26.76	129.88 ± 28.37	134.58 ± 23.36	0.381
Aortic DBP (mmHg)	78.60 ± 16.36	79.80 ± 17.86	76.26 ± 12.86	0.281
WBC (x1000/uL)	6.36 ± 2.05	6.11 ± 1.99	6.85 ± 2.10	0.071
Hemoglobin (g/dL)	10.38 ± 1.17	10.22 ± 1.25	10.70 ± 0.92	0.040*
Iron (μg/dL)	68.02 ± 26.09	66.89 ± 26.12	70.21 ± 26.24	0.526
Total iron-binding capacity (μg/dL)	263.55 ± 47.30	269.46 ± 51.62	252.05 ± 35.35	0.065
Ferritin (ng/mL)	208.50 (90.73–400.48)	221.55 (75.88–409.35)	201.70 (151.25–365.38)	0.549
Total cholesterol (mg/dL)	146.69 ± 36.21	144.68 ± 38.15	150.61 ± 32.23	0.414
Triglyceride (mg/dL)	126.50 (87.50–199.50)	108.50 (80.00–198.50)	157.00 (114.75–217.75)	0.010*
Glucose (mg/dL)	132.00 (109.25–175.75)	130.00 (106.75–163.50)	140.00 (109.75–222.00)	0.090
Blood urea nitrogen (mg/dL)	59.60 ± 13.97	59.95 ± 14.42	58.92 ± 13.21	0.715
Creatinine (mg/dL)	9.37 ± 1.90	9.46 ± 2.03	9.19 ± 1.60	0.478
Total calcium (mg/dL)	8.93 ± 0.75	8.85 ± 0.73	9.08 ± 0.77	0.133
Phosphorus (mg/dL)	4.65 ± 1.28	4.66 ± 1.37	4.64 ± 1.11	0.942
Intact parathyroid hormone (pg/mL)	204.05 (71.48–373.78)	225.50 (92.48–363.05)	165.85 (51.88–422.90)	0.452
Leptin (ng/mL)	16.53 (6.36–44.39)	8.47 (3.74–25.55)	66.08 (20.37–92.71)	<0.001*
Urea reduction rate	0.73 ± 0.05	0.73 ± 0.05	0.74 ± 0.04	0.976
Kt/V (Gotch)	1.34 ± 0.18	1.35 ± 0.19	1.34 ± 0.17	0.826

Values for continuous variables given as means ± standard deviation and test by Student’s t-test; variables not normally distributed given as medians and interquartile range and test by Mann-Whitney U test. HD, hemodialysis; SBP, systolic blood pressure; DBP, diastolic blood pressure; WBC, white blood cell; Kt/V, fractional clearance index for urea.

* P< 0.05 was considered statistically significant.

Distribution of HD patients in either high or low arterial stiffness subgroup is presented in Table 2. Therapeutic agents prescribed for our HD patients included angiotensin-converting enzyme (ACE) inhibitors, angiotensin receptor blockers (ARB), β-blockers, calcium channel blockers (CCB), statin, and fibrate were also listed. High arterial stiffness group had a higher percentage of diabetes. However, there was no statistically significant difference by subgroup analysis in gender, presence of hypertension, and use of ACE inhibitor, ARB, β-blockers, CCB, statin, or fibrate.

**Table 2:** Distribution of hemodialysis patients with high or low arterial stiffness in subgroup analysis.

Characteristics		Low arterial stiffness group (%)	High arterial stiffness group (%)	P
Gender	Male	38 (51.4)	19 (50.0)	0.892
	Female	36 (48.6)	19 (50.0)	
Diabetes	No	52 (70.3)	15 (39.5)	0.002*
	Yes	22 (29.7)	23 (60.5)	
Hypertension	No	38 (51.4)	16 (42.1)	0.354
	Yes	36 (48.6)	22 (57.9)	
ACE inhibitor use	No	71 (95.9)	38 (100)	0.208
	Yes	3 (4.1)	0 (0)	
ARB use	No	55 (74.3)	26 (68.4)	0.509
	Yes	19 (25.7)	12 (31.6)	
β-blocker use	No	52 (70.3)	23 (60.5)	0.299
	Yes	22 (29.7)	15 (39.5)	
CCB use	No	44 (59.5)	26 (68.4)	0.354
	Yes	30 (40.5)	12 (31.6)	
Statin use	No	64 (86.5)	29 (76.3)	0.175
	Yes	10 (13.5)	9 (23.7)	
Fibrate use	No	72 (97.3)	37 (97.4)	0.982
	Yes	2 (2.7)	1 (2.6)	

Data are expressed as number of patients and analysis was done using the chi-square test.

* P< 0.05 was considered statistically significant.

The association between clinical characteristics and serum leptin level is demonstrated in Table 3. There was no statistically significant difference on the leptin level by subgroup analysis in gender, presence of diabetes and hypertension, and use of ACE inhibitor, ARB, β-blockers, CCB, statin, or fibrate.

**Table 3:** Clinical characteristics and serum leptin levels of 112 hemodialysis patients.

Characteristic		Number (%)	Leptin (ng/mL)	P value
Gender	Male	57 (50.9)	12.41 (4.95–50.00)	0.341
	Female	55 (49.1)	20.57 (7.70–40.44)	
Diabetes	No	67 (59.8)	12.66 (5.11–38.15)	0.198
	Yes	45 (40.2)	19.11 (8.47–51.69)	
Hypertension	No	54 (48.2)	24.45 (7.79–56.46)	0.181
	Yes	58 (51.8)	13.66 (5.57–38.04)	
ACE inhibitor	No	109 (97.3)	19.11 (7.23–46.47)	0.051
	Yes	3 (2.7)	5.59 (2.31–5.92)	
ARB	No	81 (72.3)	16.70 (6.34–42.37)	0.915
	Yes	31 (27.7)	16.18 (6.28–51.34)	
β-blocker	No	75 (67.0)	16.37 (5.92–44.97)	0.781
	Yes	37 (33.0)	19.76 (7.05–45.30)	
CCB	No	70 (62.5)	22.26 (7.50–56.46)	0.123
	Yes	42 (37.5)	11.34 (5.44–29.79)	
Statin	No	93 (83.0)	16.18 (7.23–42.37)	0.923
	Yes	19 (17.0)	19.76 (3.71–63.48)	
Fibrate	No	109 (97.3)	16.18 (6.18–43.80)	0.127
	Yes	3 (2.7)	37.52 (33.92–91.84)	

Data are expressed as medians and interquartile range and test by Mann-Whitney U test.

ARB, angiotensin-receptor blocker; ACE, angiotensin-converting enzyme; CCB, calcium-channel blocker.

The correlation between serum leptin levels and clinical variables among 112 HD patients is depicted in Table 4. The univariate linear regression analysis revealed a positive correlation between log-leptin and post-HD body weight, waist circumference, BMI, body fat mass, cfPWV, aortic diastolic blood pressure (DBP), WBC counts and log-triglyceride in our HD patients. After multivariate stepwise linear regression analysis adjusting waist circumference, body mass index, body fat mass, carotid-femoral PWV, aortic DBP, WBC, and log-triglyceride, only body mass index, body fat mass and cfPWV were positively correlated with log-leptin level.

**Table 4:** Correlation between serum leptin levels and clinical variables among 112 hemodialysis patients.

Variables	Log-leptin (ng/mL)			
	Univariate		Multivariate	
	r	P	Beta	P
Age (years)	0.071	0.454		
Log-HD duration (years)	-0.179	0.060		
Post-HD body weight (Kg)	0.452	<0.001*		
Waist circumference (cm)	0.530	<0.001*		
Body mass index (Kg/m^2^)	0.543	<0.001*	0.300	<0.001*
Body fat mass (%)	0.608	<0.001*	0.369	<0.001*
Carotid-femoral PWV (m/s)	0.516	<0.001*	0.342	<0.001*
Aortic SBP (mmHg)	-0.167	0.078		
Aortic DBP (mmHg)	-0.235	0.013*		
WBC (x1000/uL)	0.236	0.012*		
Hemoglobin (g/dL)	0.009	0.921		
Iron (μg/dL)	0.001	0.997		
Total iron-binding capacity (μg/dL)	0.101	0.288		
Log-Ferritin (ng/mL)	-0.031	0.745		
Total cholesterol (mg/dL)	0.156	0.100		
Log-Triglyceride (mg/dL)	0.367	<0.001*		
Log-Glucose (mg/dL)	0.117	0.220		
Blood urea nitrogen (mg/dL)	-0.145	0.128		
Creatinine (mg/dL)	0.076	0.428		
Total calcium (mg/dL)	0.057	0.554		
Phosphorus (mg/dL)	0.027	0.774		
Urea reduction rate	-0.131	0.168		
Kt/V (Gotch)	--0.146	0.125-	-	-

Data of HD duration, triglyceride, glucose, ferritin, iPTH, and leptin levels showed skewed distribution, and therefore were log-transformed before analysis. Analysis data was done using the univariate linear regression analyses or multivariate stepwise linear regression analysis (adopted factors: waist circumference, body mass index, body fat mass, carotid-femoral PWV, aortic DBP, WBC, and Log-triglyceride).

*P < 0.05 was considered statistically significant.

While, after multivariable logistic regression analysis of the variables (adopted factors: diabetes, age, waist circumference, body mass index, body fat mass, hemoglobin, triglyceride, leptin, SBP and DBP) showed that diabetes (odds ratio [OR], 7.17; 95% confidence interval [CI], 1.39–37.00; p = 0.019), body fat mass (OR 1.16; CI 1.02–1.33; p = 0.027); hemoglobin (OR 2.11; CI 1.15–3.87; p = 0.015); and leptin (OR 1.09; CI 1.04–1.14; p = <0.001) were independently correlated to central arterial stiffness in our HD patients (Table 5).

**Table 5:** Multivariate logistic regression analysis of the factors correlated to arterial stiffness among 112 hemodialysis patients.

Variables	Odds ratio	95% confidence interval	p value
Diabetes	7.17	1.39–37.00	0.019*
Body fat mass	1.16	1.02–1.33	0.027*
Hemoglobin (g/dL)	2.11	1.15–3.87	0.015*
Leptin (ng/mL)	1.09	1.04–1.14	<0.001*

Analysis data was done using the multivariate logistic regression analysis (adopted factors: diabetes, age, waist circumference, body mass index, body fat mass, hemoglobin, triglyceride, leptin, systolic and diastolic blood pressure).

*P < 0.05 was considered statistically significant.

## Discussion

After adjusting age, diabetes, body mass index, body fat mass, body weight, hemoglobin, triglyceride, leptin, systolic and diastolic blood pressure in multivariable logistic regression analysis, our study found that central arterial stiffness was positively associated with leptin, diabetes, hemoglobin, and body fat mass. This finding demonstrated the coherence of hyperleptinemia and progressive arterial stiffness in ESRD patients. This association of leptin and arterial stiffness had also been shown in geriatrics, obese, or healthy population [12–14]. Moreover, our published data had delineated this association in kidney transplantation recipients [15–16].

Even the decrease of renal clearance can augment serum leptin concentration in ESRD patients [17]; however, one-quarter of chronic peritoneal dialysis patients maintained normal serum leptin level [18]. Therefore, the determinate factors of serum leptin level in ESRD subjects should be multivariate and beyond. Similar to non-dialysis population, high serum leptin level in our HD patients also corresponded with BMI and percentage of body fat mass, which support this hypothesis. An overproduction of leptin from adipose tissue may contribute to this hyperleptinemia.

Leptin had been found to able to trigger hypertension in various animal and clinical studies, thus can exacerbate atherosclerosis in cardiovascular disease [19]. Meanwhile, alpha antagonist, through the inhibition of leptin-induced sympathetic tone, was proved to reverse this high blood pressure in rat [20–21].

Before our findings, there is only one study discussed the leptin level and arterial stiffness in ESRD subjects [22]. However, this study discovered an inverse relationship between leptin and arterial stiffness, which was opposite not only to our study which focuses on HD patients, but also to the other studies on populations without kidney disease. The authors implied both acidosis status and obesity were responsible for this conflicting result. However, two questions were left to be clarified. First, among many different methods to measure arterial stiffness, only carotid-to-femoral PWV has been shown to have a predictive value for morbidity and mortality and became widely used [23]. Raikou et al. presented leptin was only inversely associated with carotid augmentation index (AIx), but not with PWV. Second, the study grouping was based on the leptin levels, instead of PWV values. The cfPWV value above 10 m/s was broadly validated with poor clinical outcome, while the cut-off point to defined hyperleptinemia was unclear.

Leptin was considered to be an antidiabetic adipokine because of its well-known effect on appetite suppression and energy regulation via thalamus. With more understanding of the function of leptin receptor in different organs, the impact of leptin is more than on energy metabolism [24]. In our HD population, more diabetic patients were in the high arterial stiffness group. The multivariate logistic regression analysis disclosed that diabetes had the strongest correlation to arterial stiffness beyond other factors. The result is consistent with the previous studies focus on non-ESRD diabetic patients [25–26].

In addition, the current study found hemoglobin level was correlated with arterial stiffness. This finding was similar to previous reports based on a large healthy Japanese cohort and on HD patients [27–29]. Possible explanations for this phenomenon are hemoglobin may activate nuclear factor-kappa B transcription, inactivate nitric oxide to mediate vascular homeostasis and hemoglobin membranes, which can induce plaque instability and atherosclerosis progression [30–32]. According to Chen's report, gender difference affects arterial stiffness in healthy Chinese population [33]. However, we failed to demonstrate this relationship in our ESRD patients. This discrepancy may due to the different study population.

## Limitation

We recognized several limitations to our analysis. The high arterial stiffness group comprised of more diabetes patients, which may confound intergroup comparisons. The categories and accumulated dosage of medications for mineral bone disease were absent. The body fat mass percentage measured before dialysis could be confounded by hypervolemic status. Moreover, the present study had a cross-sectional design and a small sample size from a single medical center. Thereby, it is beyond the scope of this study to examine the consequence of leptin on blood vessel in HD population. However, to the best of our knowledge, this study is the first one that reports a positive correlation between serum leptin level and central arterial stiffness in chronic HD patients.

## Conclusion

In conclusion, our study demonstrated that hyperleptinemia was positively correlated to the high cfPWV and thus was related to high central arterial stiffness in HD patients. Besides, a positive correlation between hemoglobin and arterial stiffness also observed.

## Supporting information

S1 Dataset (SAV)
